# Integrated ordination of miRNA and mRNA expression profiles

**DOI:** 10.1186/s12864-015-1971-9

**Published:** 2015-10-12

**Authors:** Giacomo Diaz, Fausto Zamboni, Ashley Tice, Patrizia Farci

**Affiliations:** Department of Biomedical Sciences, University of Cagliari, Cagliari, Italy; Liver Transplantation Center, Brotzu Hospital, Cagliari, Italy; Hepatic Pathogenesis Section, Laboratory of Infectious Diseases, National Institute of Allergy and Infectious Diseases, National Institutes of Health, Bethesda, MD USA

**Keywords:** MicroRNAs, mRNA, Multidimensional scaling, Gene expression, Kendall correlation, Partial correlations, Normal liver, Acute liver failure (ALF), Hepatitis B virus

## Abstract

**Background:**

Several studies have investigated miRNA and mRNA co-expression to identify regulatory networks at the transcriptional level. A typical finding of these studies is the presence of both negative and positive miRNA-mRNA correlations. Negative correlations are consistent with the expected, faster degradation of target mRNAs, whereas positive correlations denote the existence of feed-forward regulations mediated by transcription factors. Both mechanisms have been characterized at the molecular level, although comprehensive methods to represent miRNA-mRNA correlations are lacking. At present, genome-wide studies are able to assess the expression of more than 1000 mature miRNAs and more than 35,000 well-characterized human genes. Even if studies are generally restricted to a small subset of genes differentially expressed in specific diseases or experimental conditions, the number of potential correlations remains very high, and needs robust multivariate methods to be conveniently summarized by a small set of data.

**Results:**

Nonparametric Kendall correlations were calculated between miRNAs and mRNAs differentially expressed in livers of patients with acute liver failure (ALF) using normal livers as controls. Spurious correlations due to the histopathological composition of samples were removed by partial correlations. Correlations were then transformed into distances and processed by multidimensional scaling (MDS) to map the miRNA and mRNA relationships. These showed: (a) a prominent displacement of miRNA and mRNA clusters in ALF livers, as compared to control livers, indicative of gene expression dysregulation; (b) a clustering of mRNAs consistent with their functional annotations [CYP450, transcription factors, complement, proliferation, HLA class II, monocytes/macrophages, T cells, T-NK cells and B cells], as well as a clustering of miRNAs with the same seed sequence; and (c) a tendency of miRNAs and mRNAs to populate distinct regions of the MDS plot. MDS also allowed to visualize the network of miRNA-mRNA target pairs.

**Conclusions:**

Different features of miRNA and mRNA relationships can be represented as thematic maps within the framework of MDS obtained from pairwise correlations. The symmetric distribution of positive and negative correlations between miRNA and mRNA expression suggests that miRNAs are involved in a complex bidirectional molecular network, including, but not limited to, the inhibitory regulation of miRNA targets.

**Electronic supplementary material:**

The online version of this article (doi:10.1186/s12864-015-1971-9) contains supplementary material, which is available to authorized users.

## Background

MicroRNAs (miRNAs) are short non-coding RNAs that induce silencing and destabilization of messenger RNAs (mRNAs) by binding to specific target sites [1, 2]. Several studies have recently investigated miRNA and mRNA co-expression to identify post-transcriptional regulations involved in proliferative and degenerative diseases [3–8], based on the fact that the up/down-regulation of a miRNA causes the inverse down/up-regulation of its target mRNAs, and this would result in a negative correlation between miRNA and mRNA expressions. On the other hand, most studies have so far shown the co-existence of negative and positive miRNA-mRNA correlations, which are consistent with the presence of a more complex network that involves not only inhibition of miRNA targets (resulting in negative miRNA-mRNA correlations) but also feed-forward regulation activated by common transcription factors [9–11], resulting in positive miRNA-mRNA, miRNA-miRNA and mRNA-mRNA correlations. Moreover, there is increasing evidence for the existence of miRNA-miRNA [12–15] and mRNA-mRNA [16, 17] direct interactions.

A long recognized problem in correlation studies is the presence of covariates [18], which result in spurious correlations that confound the true correlations. In gene expression studies of diseases, critical covariates are represented by the different degree or extension of the histopathological lesions of samples. This is particularly relevant to human tissues, whose histological conditions are not as homogeneous as in experimental laboratory models.

Another problem concerns the visualization of data. Genome-wide studies are able to assess the expression of more than 35,000 well-characterized human genes and more than 1000 mature miRNAs. Even restricting the study to a small subset of genes differentially expressed in specific diseases or experimental conditions, the number of potential correlations is very high, and needs robust multivariate methods to be conveniently summarized by a small set of significant data [19].

These issues were addressed in this study, which was aimed at investigating the joint expression of miRNAs and mRNAs in pathologic livers obtained from patients with HBV-associated acute liver failure (ALF), a dramatic disease characterized by hepatocellular necrosis. Our previous studies in HBV-associated ALF have shown a prominent expression of B cell-related genes as well as of genes involved in liver regeneration and fibrogenesis [20, 21].

The study involved various steps. First, miRNAs and mRNAs differentially expressed in ALF were merged into a single gene-by-sample matrix. Then, partial nonparametric correlations between each gene pair (including all miRNA-mRNA, miRNA-miRNA and mRNA-mRNA combinations) were calculated to remove the effect of necrosis. Nonparametric correlations were transformed into nonmetric distances, and multidimensional scaling (MDS) was then applied to transform distances into spatial coordinates. MDS provided a comprehensive framework for thematic maps showing different features of the miRNA-mRNA, miRNA-miRNA and mRNA-mRNA interrelationships.

## Methods

### Patients and liver specimens

Thirteen liver specimens were obtained at the time of liver transplantation from 4 patients with HBV-associated ALF. The demographic, clinical, biochemical, virological and histopathological data have been previously reported [20, 21]. The control group comprised 10 liver donors and 7 subjects who underwent hepatic resection for liver angioma. Liver specimens were received under code to protect the identity of the subjects. Written informed consent was obtained from each patient or the next of kin. The study received approval by the NIH Office of Human Subjects Research, granted on the condition that all samples were made anonymous.

### RNA extraction and microarray analysis

miRNA analysis was performed using Affymetrix GeneChip miRNA 2.0 arrays (Affymetrix, Santa Clara, CA), which contain 1105 pre-miRNA (mir-), and 1105 mature miRNA (miR-) probe sets, whose nomenclature refers to miRBASE release 15 [22]. However, miRNAs removed from next miRBase releases were also excluded from the study. Total RNA was extracted from frozen liver specimens using the miRNeasy Mini Kit (Qiagen, Valencia, CA). 500 ng of total RNA, including microRNA, was poly(A)-tailed and then directly ligated to a fluorescent dendrimer (a branched single- and double-stranded DNA molecule conjugated to biotin) using the FlashTag Biotin HSR RNA Labeling Kit (Affymetrix). An ELOSA was performed prior to hybridization and analysis of the arrays in order to verify that all miRNAs were correctly labeled with the biotin molecule at the 3′ end. mRNA analysis was performed using Affymetrix Human U133 Plus 2 arrays, which contain 54,675 probe sets representing approximately 38,000 known human genes. Total RNA was extracted from frozen liver specimens using Trizol (Invitrogen, Life Technologies, Carlsbad, CA). Total liver RNA (50 ng) was subjected to two successive rounds of amplification. RNA quality and integrity were assessed with the RNA 6000 Nano Assay on the Agilent 2100 Bioanalyzer (Agilent Technologies, Santa Clara, CA). Standard Affymetrix protocols were used for hybridization, staining, washing, scanning and quality control of the arrays [23].

### Statistical analysis

Raw microarray data (cel files) were imported into BRB-ArrayTools [24] and probe set summaries were computed using the RMA algorithm. Multiple transcripts of known gene were averaged, whereas transcripts of unknown genes were discarded. Signed fold changes were calculated as the ratio between the geometric means of ALF and normal livers. A multivariate permutation F-test [25] with a maximum false discovery rate of 1 % with 80 % confidence level identified 109 miRNAs and 3239 mRNAs differentially expressed in ALF. To ensure a more robust analysis, the number of mRNAs was then reduced to 531 by selecting only mRNAs with absolute fold changes >5. The list and fold changes of miRNAs and mRNAs are shown in the Additional file 1: Tables S1-S2. Log_2_-transformed miRNAs and mRNAs expressions of ALF and control livers were merged into two separate genes × samples matrices, a 642 × 13 matrix for ALF livers, and a 642 × 17 matrix for control livers. Pairwise nonparametric partial Kendall correlations were calculated for the matrix of ALF livers, setting the degree of liver necrosis as covariate, whereas simple Kendall correlations were calculated for the matrix of normal livers, which were not affected by necrosis. The choice of a nonparametric correlation, rather than Pearson’s correlation, was forced by the fact that the expression levels of miRNAs and mRNAs, particularly in ALF samples, were not normally distributed (data not shown). Correlations were then transformed into nonmetric distances using the formula: (−1) × Kendall tau, rather than 1 - Kendall tau, to maintain the data zero-centered. The distance matrices were finally processed by MDS to obtain a dimensionally reduced map of gene coordinates. The MDS method was preferred to analogous methods (i.e., Principal Coordinates) as it allows data to be preliminarily processed by partial nonparametric correlations. MDS was computed using the singular value decomposition (SVD) method [19], which ensures a matrix factorization numerically accurate even in the presence of a high degree of multicollinearity (i.e., multiple correlation). Multivariate analyses and graphics were made using the following R functions available from the CRAN repository [26]: pcor.test {ppcor}; svd {base}; sammon, isoMDS {MASS}; ordiellipse {vegan}; ellipse3d, plot3d {rgl}; lm, density {stats}; ppp {spatstat}. An example of R code of MDS achieved using SVD is shown in [27]. MiRNA seed sequences and chromosomal loci were obtained from Affymetrix annotations to the GeneChip miRNA 2.0 [23], and verified in miRBase [22]. Only the chromosomal locus of miR-199a-3p was found in miRBase, but not in Affymetrix annotations. The identification of target mRNAs was obtained from the microRNA.org database [28], selecting miRNA-mRNA pairs with conserved miRNAs and a good (<= −0.1) mirSVR score.

## Results and discussion

### The effect of the tissue condition on spurious correlations

**Fig. 1:**
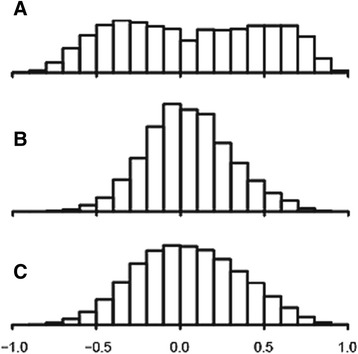
Frequency distribution comprehensive of all pairwise miRNA-mRNA, miRNA-miRNA and mRNA-mRNA Kendall correlations. **a** ALF livers. **b** normal livers. **c** ALF livers after partial correlations calculated for the level of necrosis

**Fig. 2:**
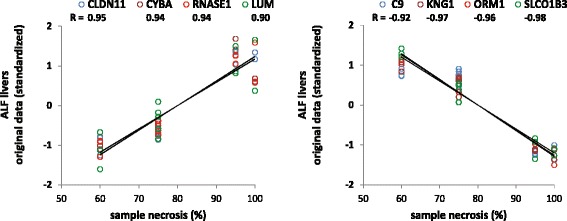
Correlation between gene expression and level of necrosis in ALF livers. The plots show a selection of representative mRNAs positively (*left*) or negatively (*right*) correlated with the degree of necrosis in ALF livers with |R| > 0.9. The level of hepatic necrosis is on the X axis; the gene expression is on the Y axis. Presumably, mRNAs positively correlated with necrosis are produced by non-hepatocyte cells, whereas those negatively correlated with necrosis are produced by hepatocytes. Gene expressions were standardized to fit the same scale range. Multiple dots of the same gene (*color*) for each level of necrosis represent data of multiple samples

In ALF livers, nonparametric correlations between each gene pair (including all miRNA-mRNA, miRNA-miRNA and mRNA-mRNA combinations) showed a prominent bimodal distribution due to a large number of highly negative and positive correlations, contrasting with the more regular, unimodal distribution of correlations of control livers (Fig. 1). This was an evident effect of the disease, as necrosis involves a loss of hepatocytes and an increase of infiltrating cells. Thus, pairs of genes prevalently expressed by hepatocytes (both ↓↓) or infiltrating cells (both ↑↑) result in spurious positive correlations, whereas pairs of genes expressed by hepatocytes and infiltrating cells (one ↓ and one ↑, respectively) result in spurious negative correlations. The impact of histopathological changes on the apparent gene expression was also indicated by the fact that about one third of genes differentially expressed in ALF livers correlated with the level of necrosis with very high correlation coefficients (|R| > 0.9). This means that more than 81 % (R squared) of the overall variability of gene expression was due to the changes in the histological composition of ALF livers. A representative sample of genes positively and negatively correlated with necrosis with |R| > 0.9 is shown in Fig. 2. We also calculated the regression between the level of necrosis (independent variable) and the mRNA concentration (dependent variables), in order to estimate the gene expression expected for zero necrosis (intercept). This involved a certain statistical licence, because zero necrosis was out of the range of data inputted in the model (some amount of necrosis is invariably present in all ALF livers).

**Fig. 3:**
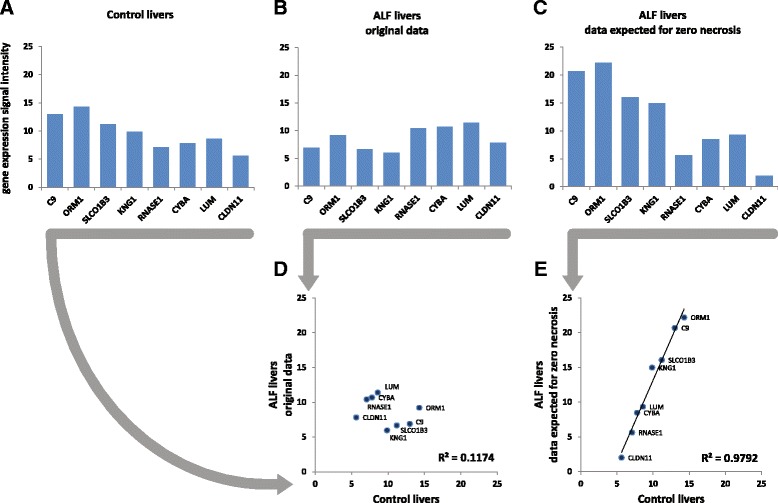
Gene expression corrected for necrosis. Data refer to the same mRNAs shown in Fig. 2. **a** control livers. **b** ALF livers. **c** ALF livers data expected for zero necrosis, obtained as the intercept of the regression between necrosis (*X variable*) and gene expression (*Y variable*). **d** correlation between control livers and original ALF livers data. **e** correlation between control livers and ALF livers data expected for zero necrosis

Using the same subset of genes as in Fig. 2, the mRNA levels of control and ALF livers, and those expected for ALF livers with zero necrosis, are shown in Fig. 3a-c. The correlation between the original mRNA levels of control and ALF livers was very low (*R* = 0.34, Fig. 3d). However, using the mRNA levels of ALF livers calculated for zero necrosis, the correlation became very high (*R* = 0.99, Fig. 3e). This finding confirmed not only that gene expressions were strongly biased by the level of necrosis, but also showed that the impact of necrosis could be effectively removed. It is also noteworthy that the analysis included both hepatocyte genes and non-hepatocyte genes (i.e., genes negatively and positively correlated with necrosis, respectively). This suggests that the opposite effects of the loss of hepatocytes and the increase of infiltrate were equally removed, thus making hepatocyte and non-hepatocyte gene expressions balanced and comparable to those of control normal livers. These preliminary findings prompted us to estimate the partial correlations for necrosis in order to investigate the genuine relationships among miRNA and mRNA gene expressions of ALF livers.

### Partial correlations and multidimensional scaling

**Fig. 4:**
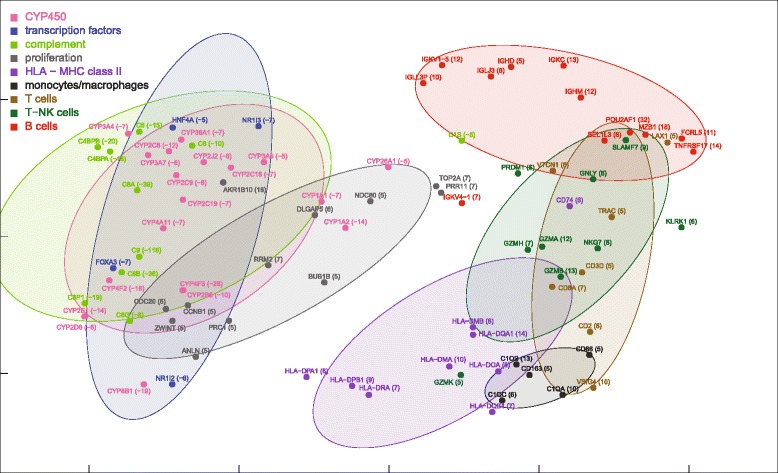
MDS mapping of mRNAs differentially expressed in ALF livers. MDS was applied to a 640 × 640 square matrix including the Kendall correlations among 109 miRNAs and 531 mRNAs differentially expressed in ALF livers. This figure shows only a subset of 87 mRNAs functionally related to CYP450, transcription factors, complement, HLA class II, monocytes/macrophages, T cells, T-NK cells and B cells. These nine functional classes are delimited by dispersion ellipses with a confidence of 1.6 standard deviations. A 360° rotation of 3D ellipses is shown in the Additional file 2: Movie 1. The nine ellipses are also shown in the next Figs. 5, 6, 7 and 8 for reference. The numbers in parentheses, on the right of gene symbols, are the fold changes of original data, not corrected for necrosis. A prominent segregation of leukocyte-related mRNAs from hepatocyte-related mRNAs is apparent

**Fig. 5:**
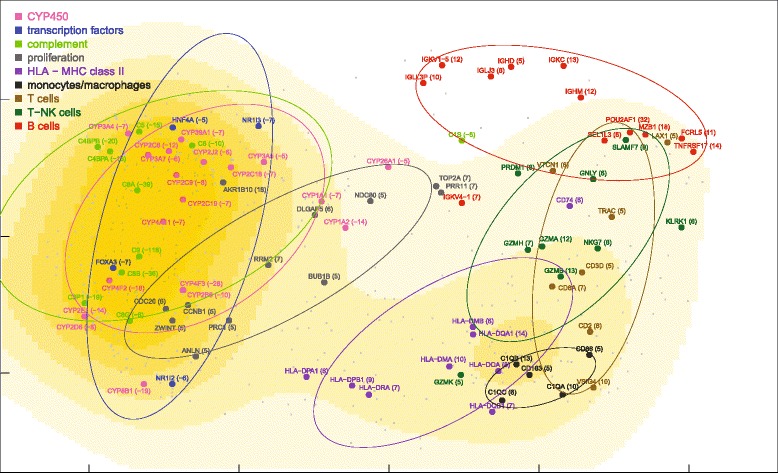
MDS density plot of mRNAs differentially expressed in ALF livers. The density plot was calculated for the MDS map of 531 mRNAs differentially expressed in ALF livers, including the 87 mRNAs of Fig. 3 (shown by large dots, symbols, original fold changes and dispersion ellipses) and the remaining 444 mRNAs (shown by small dots)

**Fig. 6:**
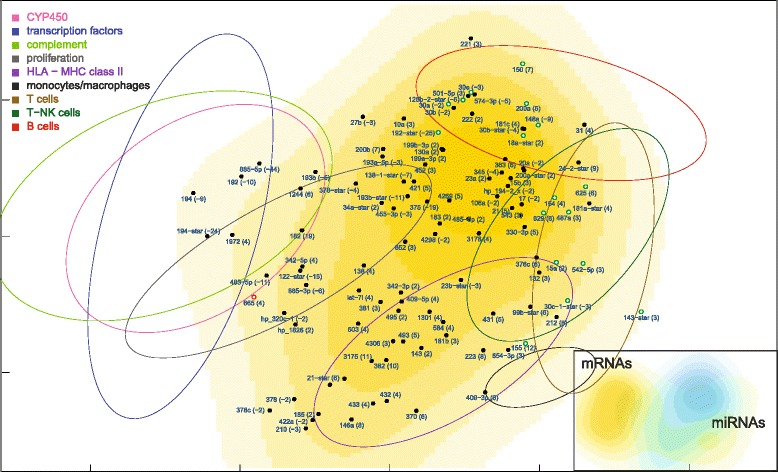
MDS density plot of miRNAs differentially expressed in ALF livers. The numbers in parentheses, on the right of miRNA symbols, are the fold changes of original data, not corrected for necrosis. The 17 green-outlined points are the miRNAs whose median correlation with mRNAs decreased more than 0.15 Kendall tau in ALF livers, whereas the red-outlined point is the only miRNA whose median correlation increased more than 0.15 Kendall tau (Additional file 4: Figure S1). The dispersion ellipses of functional mRNA clusters are shown for reference. The inset shows the complementarity of miRNA (cyan) and mRNA (yellow) MDS density plots

**Fig. 7:**
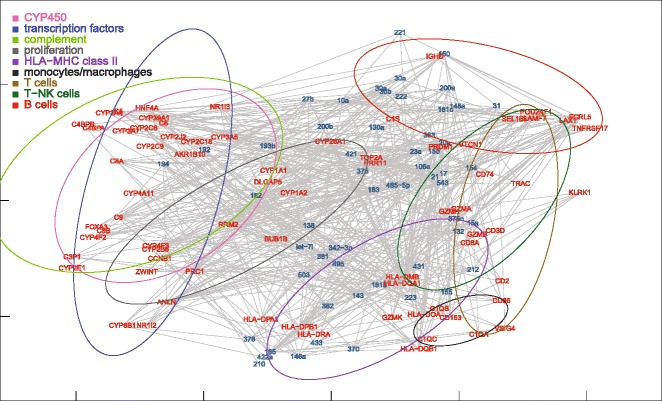
MDS mapping of the network of miRNAs and target mRNAs differentially expressed in ALF livers. For clarity, only the 87 mRNAs of the nine functional groups are shown. Target mRNAs were obtained from the microRNA.org database, selecting miRNA-mRNA pairs with conserved miRNAs and a good (<= −0.1) mirSVR score. The dispersion ellipses of functional mRNA clusters are shown for reference

**Fig. 8:**
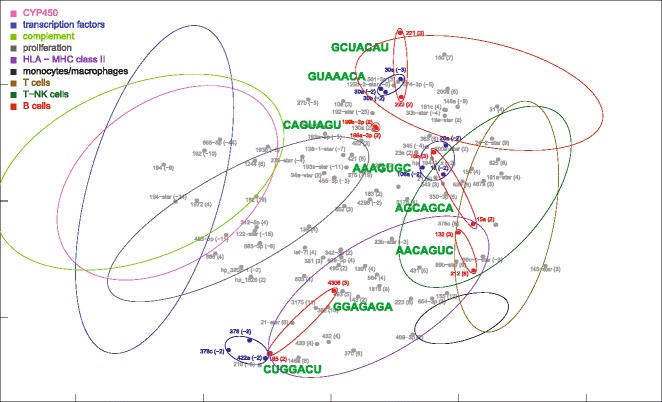
MDS mapping of miRNAs with the same seed sequence, found among miRNAs differentially expressed in ALF livers. MiRNAs with the same seed sequence are encircled by small ellipses. Small blue and red ellipses indicate down-regulated and up-regulated miRNAs, respectively. The dispersion ellipses of functional mRNA clusters are also shown for reference. The complete sequence of these miRNAs is shown in the Additional file 5: Table S3

**Fig. 9:**
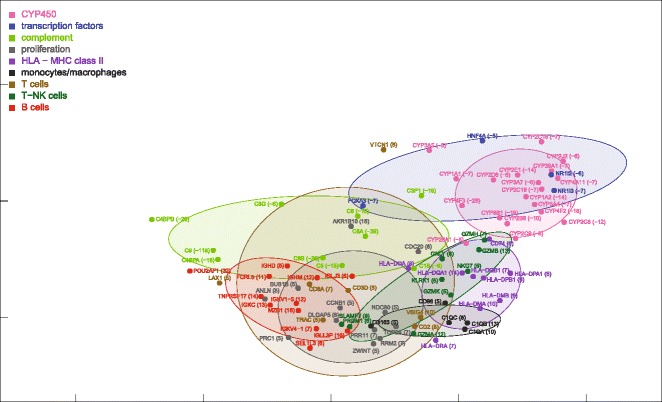
MDS mapping of mRNAs expressed in control livers. This figure is to be compared with Fig. 4. A 360° rotation of 3D ellipses is shown in the Additional file 3: Movie 2. The nine ellipses are also shown in next Figs. 10, 11, 12 and 13 for reference. The numbers in parentheses, on the right of gene symbols, are the fold changes

**Fig. 10:**
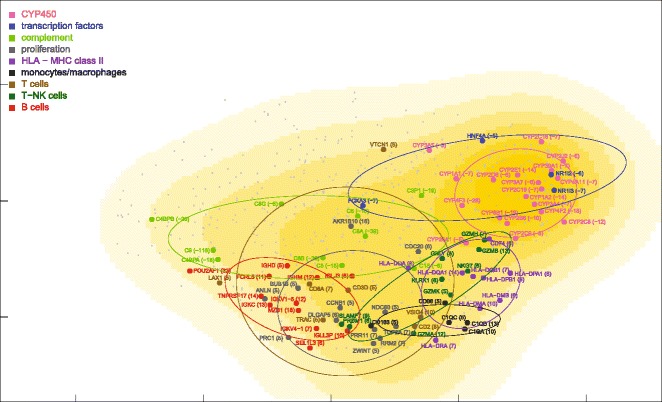
MDS density plot of mRNAs expressed in control livers. This figure is to be compared with Fig. 5

**Fig. 11:**
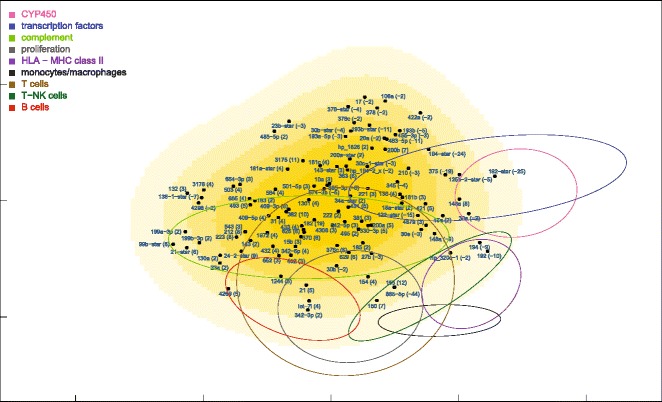
MDS density plot of miRNAs expressed in control livers. This figure is to be compared with Fig. 6

**Fig. 12:**
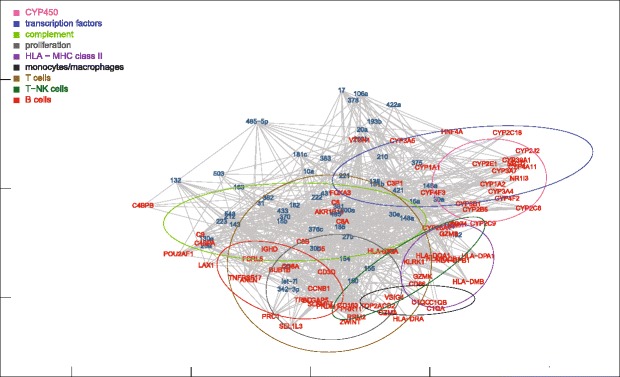
MDS mapping of the network of miRNAs and target mRNAs expressed in control livers. This figure is to be compared with Fig. 7

**Fig. 13:**
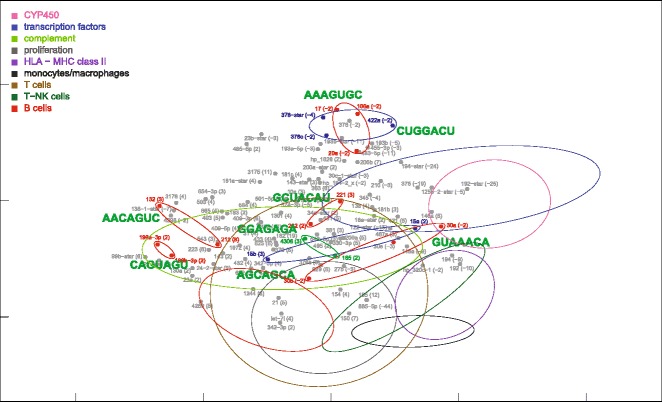
MDS mapping of miRNAs with the same seed sequence expressed in control livers. This figure is to be compared with Fig. 8

Partial nonparametric (Kendall) correlations of ALF livers showed a unimodal distribution, similar, although somewhat flatter, to that of control livers (Fig. 1c). Partial correlations were transformed into distances and then processed by MDS using the singular value decomposition method. MDS provided a general framework for thematic maps showing different features of miRNA-mRNA, miRNA-miRNA and mRNA-mRNA interrelationships (Figs. 4, 5, 6, 7 and 8 for ALF livers; Figs. 9, 10, 11, 12 and 13 for control livers). Though all 531 mRNAs differentially expressed in ALF livers were included in the analyses, for reasons of clarity only the symbols of a subset of 87 mRNAs, attributable to 9 well-defined functional groups (CYP450, transcription factors, complement, proliferation, HLA class II, monocytes/macrophages, T cells, T-NK cells and B cells, whose genes are listed in Table 1) are shown in MDS maps. The remaining mRNAs are graphically represented by points.

**Table 1:** Functional classes of genes

CYP family
CYP8B1 (-19), CYP4F3 (-28), CYP4F2 (-18), CYP4A11 (-7), CYP3A7 (-6), CYP3A5 (-5), CYP3A4 (-7), CYP39A1 (-7), CYP2J2 (-6), CYP2E1 (-14), CYP2D6 (-8), CYP2C9 (-8), CYP2C8 (-12), CYP2C19 (-7), CYP2C18 (-7), CYP2B6 (-10), CYP26A1 (-5), CYP1A2 (-14), CYP1A1 (-7)
Transcription factors
NR1I3 (-7), NR1I2 (-6), HNF4A (-5), FOXA3 (-7)
HLA class II
HLA-DRA (7), HLA-DQB1 (7), HLA-DQA1 (14), HLA-DPB1 (9), HLA-DPA1 (6), HLA-DOA (8), HLA-DMB (6), HLA-DMA (10), CD74 (6)
B cells
TNFRSF17 (14), SEL1L3 (6), POU2AF1 (32), MZB1 (18), IGLL3P (10), IGLJ3 (6), IGKV4-1 (7), IGKV1-5 (12), IGKC (13), IGHM (12), IGHD (5), FCRL5 (11)
Monocytes/macrophages
CD86 (5), CD163 (5), C1QC (6), C1QB (13), C1QA (10)
Cell proliferation
ZWINT (5), TOP2A (7), RRM2 (7), PRR11 (7), PRC1 (5), NDC80 (5), DLGAP5 (6), CDC20 (6), CCNB1 (5), BUB1B (5), ANLN (5), AKR1B10 (18)
T cells
VTCN1 (6), VSIG4 (10), TRAC (5), LAX1 (5), CD8A (7), CD3D (5), CD2 (8)
T-NK cells
SLAMF7 (9), RASGRP1 (7), PRDM1 (6), NKG7 (6), KLRK1 (6), GZMK (5), GZMH (7), GZMB (13), GZMA (12), GNLY (6)
Complement^a^
C9 (-118), C8G (-6), C8B (-36), C8A (-39), C6 (-10), C5 (-15), C4BPB (-20), C4BPA (-18), C3P1 (-19), C1S (-6)

In parentheses are the fold changes of original data, not corrected for necrosis

^a^Complement components C1QC, C1QB and C1QA were attributed to monocytes/macrophages as these proteins are mostly produced by these cells

### miRNA and mRNA co-expression maps

ALF livers showed a clear segregation of leukocyte-related mRNAs (HLA class II, monocytes/macrophages, T cells, T-NK cells and B cells) from hepatocyte-related mRNAs (CYP450, transcription factors, complement) (Fig. 4), at variance with normal liver mRNAs which were densely interconnected (Fig. 9). A 360° rotation of the MDS maps of these mRNAs in ALF and control livers is shown in Additional files 2 and 3: Movies 1–2. A clustered arrangement of the five groups of leukocyte-related mRNAs was also evident in ALF livers, in spite of the wider overall spreading. A major overlap was found between T and T-NK cell mRNAs, consistent with the strong functional interrelation of T and NK cells. Interestingly, MDS enabled the identification of a unique relationship between T and T-NK cell mRNAs and B cell mRNAs in correspondence of regulatory genes, but not of Ig genes, which encode the terminal effectors of humoral immunity. In addition, B cell mRNAs were located in a region characterized by the lowest mRNA density (Fig. 5). This suggests that B cell mRNAs were those which deviated more markedly from the configuration of normal livers. Paradoxically, the region with the lowest density of mRNAs was also the one with the highest density of miRNAs, revealing a sort of complementarity of miRNA and mRNA maps on a large scale (Fig. 6, inset). A similar complementarity was also seen in normal livers (Fig. 11). Other nonmetric MDS methods such as Kruskal’s MDS and Sammon’s mapping [29, 30] produced MDS maps substantially similar to those obtained using Kendall correlation. On the other hand, less comparable patterns were obtained using MDS based on metric (i.e., Euclidean) distances.

### Numerical comparison of miRNA-mRNA relationships in ALF and control livers

To compare numerically the miRNA-mRNA relationships in ALF and control livers, for each miRNA we computed the median correlation between that miRNA and all mRNAs (Additional file 4: Figure S1). Using an arbitrary threshold of ± 0.15 Kendall tau, a decreased correlation was found in 17 miRNAs (miR-143-star, 625, 542-5p, 30c-1-star, 18a-star, 200a, 629, 150, 125b-2-star, 30e, 155, 154, 192-star, 30a, 15a, 487a, 148a), all located within or very close to the B and T cell mRNAs in the MDS map of ALF livers (shown as green-outlined points in Fig. 6). Conversely, an increased correlation was found only in a single miRNA (miR-665), located on the opposite side of the MDS map (shown as a red-outlined point in Fig. 6). The discrepancy between these two findings is in agreement with the inhibitory effect of miRNAs.

### MDS location of miRNA-mRNA target pairs

We also mapped miRNA-mRNA target pairs (Figs. 7 and 12). In general, miRNAs and target mRNAs were located far apart from each other. In view of the fact that the distance in the MDS plot accounts for a negative correlation, this finding appeared to be suggestive of the inhibitory relationship between miRNA-mRNA target pairs. To test this hypothesis, we simulated an alternative MDS plot by inverting the sign of miRNA-mRNA correlations. Surprisingly, the average distance between miRNAs and their target mRNAs was unchanged. This means that the distance between single miRNA-mRNA pairs does not reflect only the negative regulation of miRNA targets, but also positive feed-forward co-expressions mediated by transcription factors [9–11]. This hypothesis is consistent with the symmetric distribution of positive and negative miRNA-mRNA correlations observed in this and previous studies [3–7, 31–33]. On the other hand, it must be also considered that the 2D MDS map does not exhaust the whole multidimensional structure of data. We therefore performed a multiple regression between the mirSVR score, a conventional estimate of mRNA down-regulation calculated for each miRNA-mRNA pair [28], and the distance between the same miRNA-mRNA pair in each of the first 50 MDS dimensions. This analysis showed a statistically significant relationship (*p* = 0.032), although predictively poor (multiple R-squared = 0.131), between mirSVR scores and MDS distances. By contrast, the same test performed on control livers was not statistically significant. This may be attributed to the fact that the miRNAs and mRNAs under investigation were those differentially expressed in ALF livers.

### MDS location of miRNAs with the same seed sequence

Finally, we also mapped 8 groups of miRNAs which showed the same seed sequence (Figs. 8 and 13). The complete sequence and chromosomal origin of these miRNAs is reported in the Additional file 5: Table S3. Some miRNAs were similar through their entire sequence, differing by just one or two bases. Two miRNAs in particular (199a-3p and 199b-3p) were perfectly identical despite the different chromosomal origin; thus that their distance, however small, could be ascribed to purely technical factors. On the other hand, other miRNAs (i.e., miR-221 and 222; miR-30b and 30a/30e) showed several differences in the base sequence. Interestingly, in ALF livers these miRNAs were located in the B-cell region.

### Knowledge-based and knowledge-independent methods

The functional characterization of a set of differentially expressed genes is generally based on enrichment analysis [34] using Gene Ontology [35] and/or other gene annotations. A major limit of these methods is that they depend on the knowledge publicly available at the time of the investigation. This is particularly relevant, for example, for miRNAs whose list is continuously growing (known mature human miRNAs are at present 2588, but they were only 313 about ten years ago), and the number of miRNA targets experimentally validated is only a minimal fraction (less than 2 %) of those numerically predicted. Alternatively, gene expressions can be investigated using knowledge-independent methods. One of the most used methods is hierarchical clustering, often associated with heat maps. On the other hand, hierarchical clustering methods are strongly sensitive to the linkage method adopted and, in addition, their standard output, the tree diagram, is not adequate to represent a relational network. A more suitable but less used method is MDS, which allows a number of useful options such as the preliminary control of covariates and the adoption of nonmetric distances, less sensitive to nonlinear relationships.

### Hidden covariates and confounding factors

The problem of covariate interference is of great importance in correlations studies, in particular when large (‘omics’) data sets are investigated from tissues affected by pathological alterations, including, but not limited to, tumors, inflammation, necrosis and fibrosis, present with different severity in different samples, and whenever gene expression profiles are associated to phenotypic traits [36]. In this study, it was relatively easy to recognize necrosis as a confounding covariate, as samples where histologically characterized. We have shown that removing the effect of necrosis (re)establishes a linear relationship between gene expressions of control and ALF livers, including both hepatocyte and non-hepatocyte-related genes (Figs. 2 and 3). However, it is very likely that other latent covariates may exist, possibly associated with the origin of ALF, rather than with its final outcome resulting in necrosis. But the biological factors and genetic predisposition involved in ALF pathogenesis are still largely unknown. A drastic alternative would be that of calculating partial correlations ‘within’ genes (i.e., each gene versus all others, as in some prediction-oriented methods), but this would also remove the genuine interactions at the molecular level, representing gene co-regulations.

### Emerging evidences for a complex regulatory network

mRNA destabilization induced by miRNAs has been successfully demonstrated in strictly controlled experimental conditions by hyper-expressing or silencing a single or a few miRNAs at a time [37, 38], but this is less achievable in observational studies, due to the simultaneous presence of a high number of genes differentially expressed. On the other hand, the balanced number of positive and negative miRNA-mRNA correlations observed in this and previous studies [3–7, 31–33] is consistent with the presence of a complex network involving not only the inhibitory regulation of miRNA-targeted mRNAs, but also feed-forward regulations of both miRNAs and mRNAs, activated by common transcription factors [9–11], as well as miRNA-miRNA [12–15] and mRNA-mRNA [16, 17] direct interactions.

## Conclusions

The symmetric distribution of positive and negative correlations between miRNA and mRNA expression suggests that miRNAs are involved in a complex bidirectional molecular network including, but not limited to, the inhibitory regulation of miRNA targets. Different features of this network can be represented as thematic maps within the framework of a MDS analysis applied to the whole set of pairwise correlations. MDS made it possible to visualize: (a) a prominent displacement of miRNAs and mRNAs in ALF livers, indicative of gene expression dysregulation; (b) a clustering of mRNAs consistent with their functional annotation; (c) a tendency of miRNAs and mRNAs to populate distinct regions of MDS; (d) a map of miRNA-mRNA target pairs.

## Availability of supporting data

MicroRNA and mRNA microarray data sets supporting the results of this article are available in Gene Expression Omnibus at http://www.ncbi.nlm.nih.gov/geo/query/acc.cgi?acc=GSE62037.

## Additional files

Additional file 1: Table S1. List of 109 miRNAs differentially expressed in patients with HBV-associated acute liver failure (ALF). **Table S2.** List of 531 mRNAs differentially expressed in patients with HBV-associated acute liver failure (ALF). (XLSX 37 kb) Additional file 2: Movie 1. 360° rotation of 3D MDS dispersion ellipses of mRNA functional clusters of ALF livers. Labels are shown in the two last frames (38–39) at the end of the rotation cycle. If the media player runs continuously, it should be stopped manually to visualize the labels. The original frame size is 731x712. The player window should be properly resized to optimize the image quality. (SWF 1150 kb) Additional file 3: Movie 2. 360° rotation of 3D MDS dispersion ellipses of mRNA functional clusters of control livers. Labels are shown in the two last frames (38–39) at the end of the rotation cycle. If the media player runs continuously, it should be stopped manually to visualize the labels. The original frame size is 731x712. The player window should be properly resized to optimize the image quality. (SWF 1101 kb) Additional file 4: Figure S1. Median Kendall correlations between each miRNA and all mRNAs, in normal and ALF livers. The vertical distance between each data point and the diagonal (equality line) indicates the correlation change. An arbitrary threshold of ±0.15 Kendall tau is traced by the dashed lines parallel to the main diagonal. Based on this threshold, seventeen miRNAs (green symbols) show a decreased correlation in ALF livers, while only one miRNA (red symbol) shows an increased correlation. (PDF 258 kb) Additional file 5: Table S3. MiRNAs with the same seed sequence, found among the 109 miRNAs differentially expressed in patients with HBV-associated acute liver failure (ALF). (XLSX 12 kb)
