# Supplementary figures and images for: Integrated ordination of miRNA and mRNA expression profiles
Source: BMC Genomics. 2015 Oct 12;16:767. doi: 10.1186/s12864-015-1971-9 (PMC4603994; doi:10.1186/s12864-015-1971-9)

Figure S1

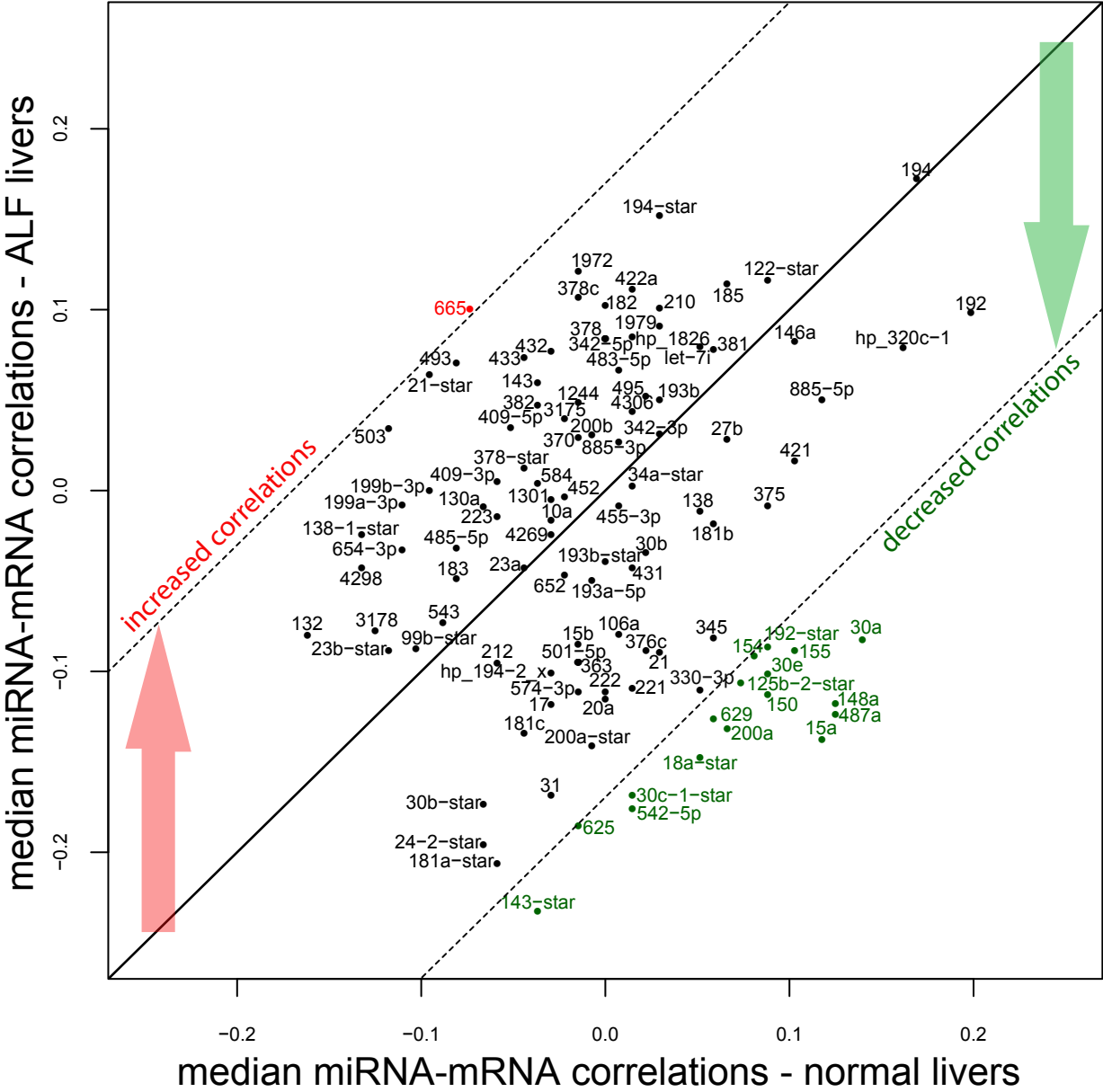

Supplement: Additional file 4: Figure S1. — Median Kendall correlations between each miRNA and all mRNAs, in normal and ALF livers. The vertical distance between each data point and the diagonal (equality line) indicates the correlation change. An arbitrary threshold of ±0.15 Kendall tau is traced by the dashed lines parallel to the main diagonal. Based on this threshold, seventeen miRNAs (green symbols) show a decreased correlation in ALF livers, while only one miRNA (red symbol) shows an increased correlation. (PDF 258 kb) [file 12864_2015_1971_MOESM4_ESM.pdf]
